# Domain architecture divergence leads to functional divergence in binding and catalytic domains of bacterial and fungal cellobiohydrolases

**DOI:** 10.1074/jbc.RA120.014792

**Published:** 2020-08-18

**Authors:** Akihiko Nakamura, Daiki Ishiwata, Akasit Visootsat, Taku Uchiyama, Kenji Mizutani, Satoshi Kaneko, Takeshi Murata, Kiyohiko Igarashi, Ryota Iino

**Affiliations:** ^1^Department of Applied Life Sciences, Faculty of Agriculture, Shizuoka University, Shizuoka, Shizuoka, Japan; 2 Department of Functional Molecular Science, School of Physical Sciences, SOKENDAI (The Graduate University for Advanced Studies), Hayama, Kanagawa, Japan; 3 Institute for Molecular Science, National Institutes of Natural Sciences, Okazaki, Aichi, Japan; 4 Department of Biomaterials Sciences, Graduate School of Agricultural and Life Sciences, University of Tokyo, Tokyo, Japan; 5 Graduate School of Medical Life Science, Yokohama City University, Tsurumi, Yokohama, Japan; 6 Department of Subtropical Biochemistry and Biotechnology, Faculty of Agriculture, University of the Ryukyus, Nishihara, Okinawa, Japan; 7 Department of Chemistry, Graduate School of Science, Chiba University, Inage, Chiba, Japan

**Keywords:** cellulase, cellulose, single-molecule observation, Cellulomonas fimi, cellulase, carbohydrate-binding protein, single-molecule biophysics, microscopic imaging, molecular imaging, glycoside hydrolase, processivity, glycoside hydrolase family 6, Trichoderma reesei

## Abstract

Cellobiohydrolases directly convert crystalline cellulose into cellobiose and are of biotechnological interest to achieve efficient biomass utilization. As a result, much research in the field has focused on identifying cellobiohydrolases that are very fast. Cellobiohydrolase A from the bacterium *Cellulomonas fimi* (CfCel6B) and cellobiohydrolase II from the fungus *Trichoderma reesei* (TrCel6A) have similar catalytic domains (CDs) and show similar hydrolytic activity. However, TrCel6A and CfCel6B have different cellulose-binding domains (CBDs) and linkers: TrCel6A has a glycosylated peptide linker, whereas CfCel6B's linker consists of three fibronectin type 3 domains. We previously found that TrCel6A's linker plays an important role in increasing the binding rate constant to crystalline cellulose. However, it was not clear whether CfCel6B's linker has similar function. Here we analyze kinetic parameters of CfCel6B using single-molecule fluorescence imaging to compare CfCel6B and TrCel6A. We find that CBD is important for initial binding of CfCel6B, but the contribution of the linker to the binding rate constant or to the dissociation rate constant is minor. The crystal structure of the CfCel6B CD showed longer loops at the entrance and exit of the substrate-binding tunnel compared with TrCel6A CD, which results in higher processivity. Furthermore, CfCel6B CD showed not only fast surface diffusion but also slow processive movement, which is not observed in TrCel6A CD. Combined with the results of a phylogenetic tree analysis, we propose that bacterial cellobiohydrolases are designed to degrade crystalline cellulose using high-affinity CBD and high-processivity CD.

Cellobiohydrolases (CBHs) play key roles in degradation of crystalline cellulose, which is the homopolymer of β-1,4-linked glucose and fundamental component of plant cell wall (1). High hydrolytic activity of CBHs against crystalline cellulose is achieved by the unique structure of the catalytic domain (CD), which consists of tunnel-shaped substrate-binding sites covered by loops (2). In addition, many CBHs also have the cellulose-binding domain (CBD), and the CD and CBD are connected by the linker region (or domain). The CD and CBD are classified into glycoside hydrolase (GH) and carbohydrate binding module (CBM) families, respectively, according to the amino acid sequences (3). Although cellulases are classified into GH families 5–12, 44, 45, 48, 51, 74, 124, and 148, CBHs are only included in the members of GH6, 7, 9, and 48. The CBMs, which have flat surfaces for cellulose binding (called as type A CBM), are divided into CBM 1, 2, 3, 5, and 10 (4).

Cellulose is the most abundant biomass on earth and an important carbon source for fungi and bacteria. Cellulose degradation system of fungi has been well-known, and they produce many kinds of multidomain cellulases. Synergistic hydrolytic reactions between GH7 and GH6 CBHs (5) or CBHs and endoglucanases (EGs) have been studied in detail. An important cellulose degradation system of bacteria is cellulosome, which is the large complex of carbohydrate active enzymes anchored to the cell surface. The cellulosome system is employed by anaerobic bacteria, and only GH5, 8, 9, and 48 cellulases are reported as components (6). Another degradation system of bacteria is similar to the fungal one. For example, an actinomycetes *Cellulomonas fimi* produces free GH6 and GH48 CBHs (7).

In the process of crystalline cellulose hydrolysis, cellulases first bind on the cellulose surface. However, after the binding, not all cellulases can initiate hydrolysis, because accessible position is limited because of the tight packing of the cellulose chains in the crystal. When a cellulase molecule successfully catches a cellulose chain into the catalytic site, it can form productive complex. In other cases, cellulase binds nonproductively and then dissociates from the cellulose surface without hydrolysis. The unique function of CBH is a unidirectional movement on cellulose surface coupled with processive hydrolysis of the cellulose chain into cellobiose, the minimum repeating unit. The unidirectional movement of CBH has been directly observed by single-molecule imaging techniques recently. For the GH7 CBHs, which is a unique cellulase for fungi, the movement was first proved by high-speed atomic force microscopy (8, 9). Furthermore, the relationship between lengths of the tunnel-like structure of the CD and processivity has been analyzed experimentally and theoretically (10, 11). Although GH6 CBHs are common enzymes in fungi and bacteria, the movement of GH6 from only an ascomycete *Trichoderma reesei* (TrCel6A) has been observed by single-molecule fluorescence imaging (12). Because GH7 CBH hydrolyzes cellulose from reducing end and GH6 CBH hydrolyzes from nonreducing end of the cellulose chain, they show opposite directionality in the processive movements.

GH6 CBHs from fungi and bacteria are classified into the same family but are different in many points. First, the tunnel-like structure of the substrate-binding site of bacterial CD is longer than that of fungi CD (13). Therefore, bacterial GH6 CBH was expected to be more processive and less endolytic than fungal GH6 CBH. Second, the CBD and linker region are different. TrCel6A has CBM1-CBD connected to CD by a glycosylated linker region (Fig. 1*A*). The linker region is expected to be intrinsically disordered, and the interaction of sugars on the linker with the cellulose surface is investigated by the molecular dynamics simulation (14). In contrast, GH6 CBH from the bacterium *C. fimi* (CfCel6B) has CBM2-CBD and three fibronectin type 3 domains (FN3s) as a linker between CD and CBD. Both CBDs have flat surface with hydrophobic residues and are expected to bind on the hydrophobic surface of the crystalline cellulose (15, 16). CfCel6B is previously called CbhA and found as the first enzyme that is similar to the CBH II from *T. reesei* (another name of TrCel6A) (17). However, the effects of the different domain composition on the elementary steps of cellulose hydrolysis reaction are still elusive.

**Figure 1.**
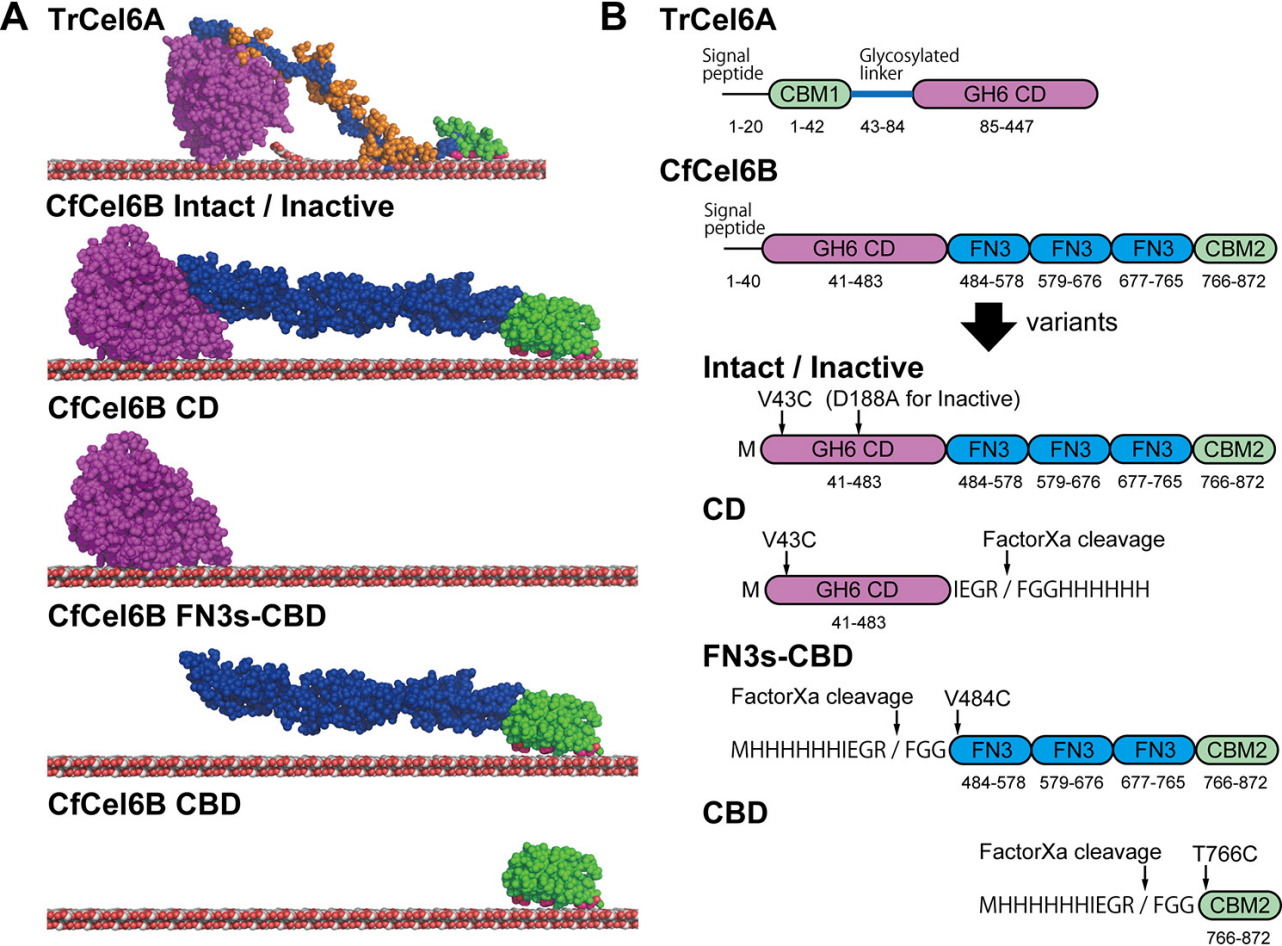
**Structures of TrCel6 and CfCel6B.** *A*, model structures of Intact TrCel6A, CfCel6B, and CfCel6B domain constructs used in this study. TrCel6A structure is the same as in the previous report (12). For CfCel6B, structure of CD is X-ray crystal structure (PDB code 7CBD), and FN3s and CBD are modeled by SWISS-MODEL server (38). Figures were prepared by PyMOL. *B*, detailed descriptions of domain compositions for each construct. Positions of mutation sites, histidine tags, and FaXa cleavage sites and estimated amino acid numbers for each domain are shown.

In this study, by using single-molecule fluorescence imaging, we observed binding and dissociation of full-length CfCel6B and its domains (CD, CBD, and FN3s-CBD) on the crystalline cellulose to clarify the functions of CBD and FN3s. Furthermore, their movements on the crystalline cellulose were analyzed. Combined with crystal structures of the CD from bacterial and fungal GH6 CBHs (CfCel6B and TrCel6A, respectively), we successfully verified correlation between lengths of the tunnel-like structure of the CD and processivity. Furthermore, in our phylogenetic tree analysis, the CDs from bacteria and fungi were clearly separated. The fungal CBHs have glycosylated linker and CBM1, and bacterial CBHs have CBM2 except for the single domain enzymes. Given the difference of domain compositions between bacterial and fungal CBHs, CBM2 of bacterial CBHs compensates for facilitated initial binding on cellulose by glycosylated linkers of fungal CBHs. Our results indicate the difference of a design principle between bacterial CBHs and fungal CBHs.

## Results

### Preparation of fluorescently labeled samples

**Figure 2.**
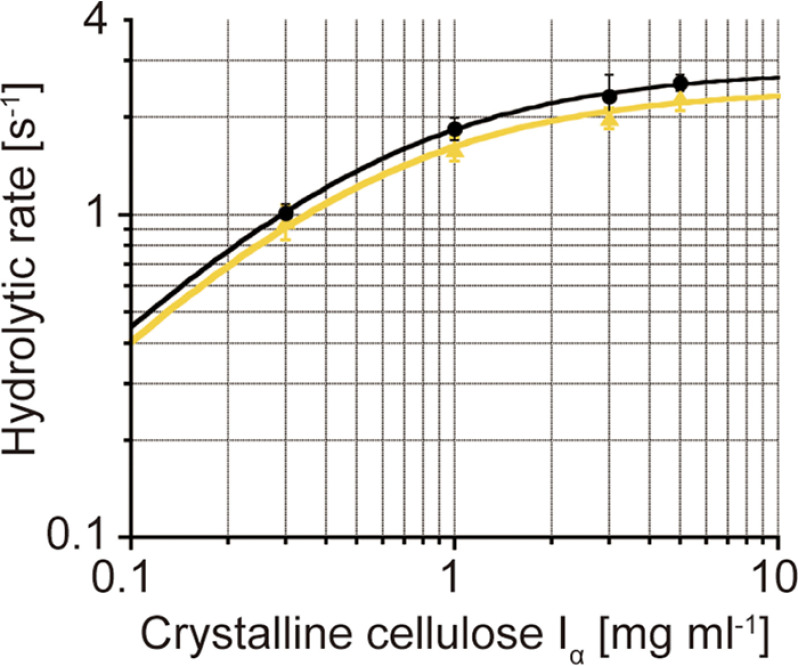
**Michaelis–Menten plots of CfCel6B WT and Intact.** Hydrolytic rates of WT (*black circles*) and Intact (*yellow triangles*) at various crystalline cellulose concentrations (0.3, 1.0, 3.0, and 5.0 mg ml^−1^) were fitted by Michaelis–Menten equations. The values of *K_m_* for WT and Intact were 0.52 and 0.51 mg ml^−1^, respectively. The values of *k*_cat_ for WT and Intact were 2.8 and 2.4 s^−1^, respectively. *R*^2^ values of the fitting for WT and Intact were 0.98 and 0.99, respectively. Enzyme concentration was 100 nM.

In this study, to conduct single-molecule fluorescence imaging, single free cysteines were introduced on the surface of full-length CfCel6B and its domains. We prepared full-length CfCel6B/V43C (termed as Intact), CD/V43C (CD), FN3s-CBD/V484C (FN3s-CBD), and CBD/T766C (CBD) (Fig. 1*A*). In addition, a catalytically inactive D188A mutant of full-length CfCel6B/V43C (Inactive), of which catalytic acid aspartate was mutated to alanine, was prepared as a negative control of processive movement coupled with catalysis (Fig. 1*B*). All of proteins were successfully expressed in *Escherichia coli* and purified using cellulose affinity column or nickel–nitrilotriacetic acid affinity column. After the labeling of the free cysteine with Cy3-maleimide, hydrolytic activities of Cy3-labeled Intact and Inactive were compared with WT CfCel6B. 500 nM WT hydrolyzed 1 mg ml^−1^ crystalline cellulose I_α_ at the rate of 0.068 ± 0.001 s^−1^, and Intact showed comparative hydrolytic rate (0.059 ± 0.001 s^−1^). On the other hand, hydrolytic rate of Inactive was very low (0.0080 ± 0.0010 s^−1^), indicating that Inactive do not have cellulose hydrolytic activity. Then we further compared hydrolytic rates for WT and Intact at various concentrations of crystalline cellulose I_α_ to determine the *K_m_* and *k*_cat_ values by the fitting with Michaelis–Menten equations (Fig. 2). The plots were fitted well (*R*^2^ values for WT and Intact were 0.98 and 0.99, respectively); *k*_cat_ and *K_m_* for WT were 2.8 s^−1^ and 0.52 mg ml^−1^, and those for Intact were 2.4 s^−1^ and 0.51 mg ml^−1^, respectively. Note that hydrolytic activity was also dependent on enzyme concentration, and 100 nM enzymes were used for these measurements. These results indicated that V43C mutation and labeling with Cy3 do not significantly affect the hydrolytic activity and affinity of CfCel6B against crystalline cellulose.

### Binding rate constant

**Figure 3.**
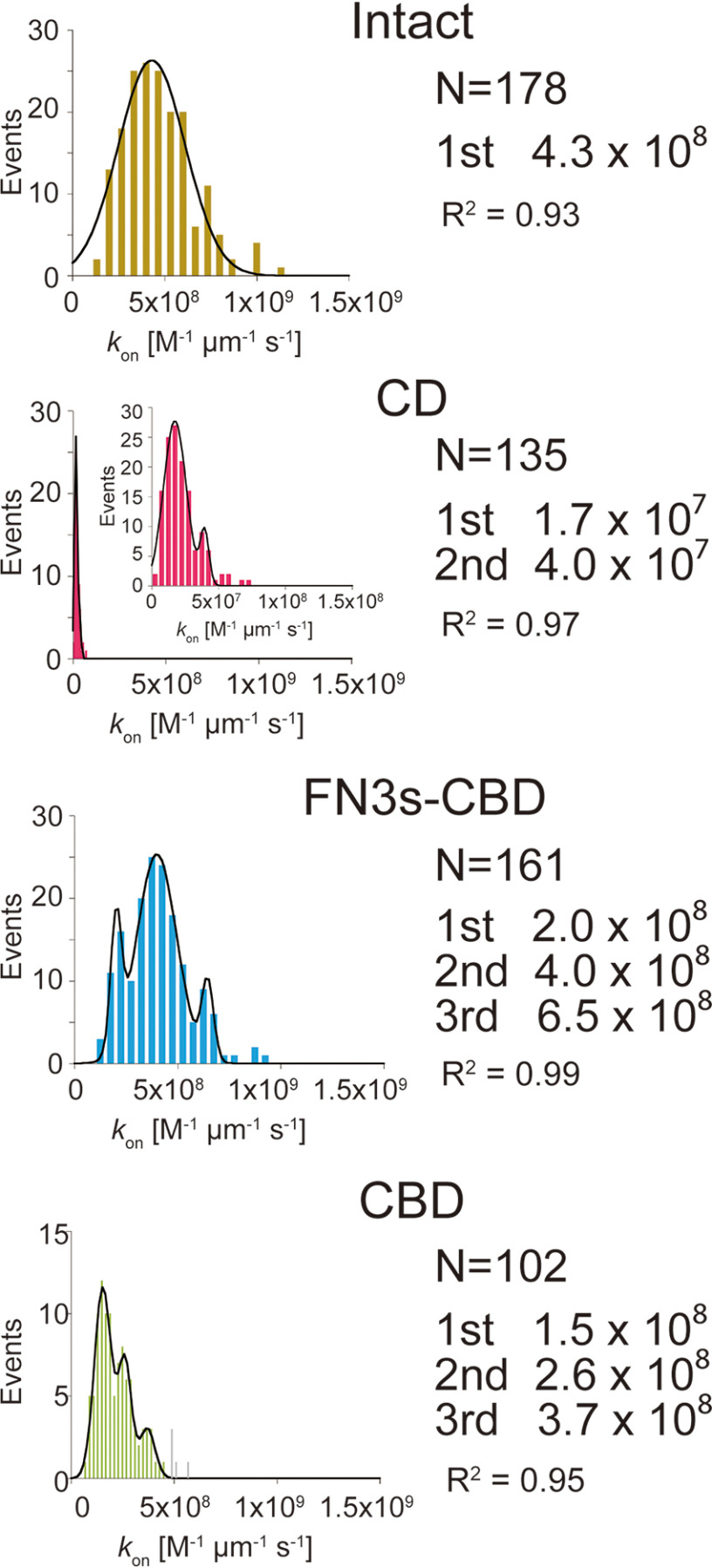
**Binding rate constants (*k*_on_) analysis of Intact, CD, FN3s-CBD, and CBD of CfCel6B.** Distributions of *k*_on_ were fitted by Gaussian functions. Peak values of Gaussian fitting are shown in right. The *N* values represent numbers of cellulose microfibrils analyzed.

To determine the binding rate constant (*k*_on_) for Intact, CD, FN3s-CBD, and CBD, enzymes of picomolar concentrations were dropped on the cover glass sparsely coated with crystalline cellulose microfibrils to clearly observe fluorescence signals from individual molecules. The bindings of enzymes were highly specific to the cellulose microfibrils, and almost no nonspecific bindings to the glass surface were observed. These results indicate that all of the enzymes have correctly folded structures that recognize the surface of crystalline cellulose. The values of *k*_on_ were calculated as numbers of bound molecules divided by enzyme concentration, length of cellulose microfibril, and observation time (m^−1^ μm^−1^ s^−1^). We could not directly estimate the number of bundles in the cellulose microfibrils from the fluorescence image stained with nanomolar concentrations of enzymes, because of the limit of spatial resolution of optical microscopy. Therefore, we analyzed the distributions of *k*_on_ (Fig. 3). Distribution of *k*_on_ for Intact showed single peak at 4.3 × 10^8^ m^−1^ μm^−1^ s^−1^. Distributions of *k*_on_ for CD, FN3s-CBD, and CBD showed multiple peaks, which would correspond to number of bundles in cellulose microfibrils. The distributions were fitted well, and their *R*^2^ values were better than 0.93. The smallest peak values for FN3s-CBD and CBD were 2.0 × 10^8^ and 1.5 × 10^8^ m^−1^ μm^−1^ s^−1^, respectively. These values were almost half and one-third of that for Intact. On the other hand, distribution of *k*_on_ for CD showed a smallest peak at 1.7 × 10^7^ m^−1^ μm^−1^ s^−1^, which was less than one-twentieth of that for Intact. These results indicate that *k*_on_ of CfCel6B is highly dependent on the binding of the CBD, and FN3s and CD do not contribute significantly. However, if CD and CBD were connected by FN3s, these two domains seem to bind synergistically, because the *k*_on_ value for Intact is larger than the simple sum of those for CD and FN3s-CBD. Synergistic binding between CBD with linker region and CD has been also observed in TrCel6A (12).

### Dissociation rate constant

**Figure 4.**
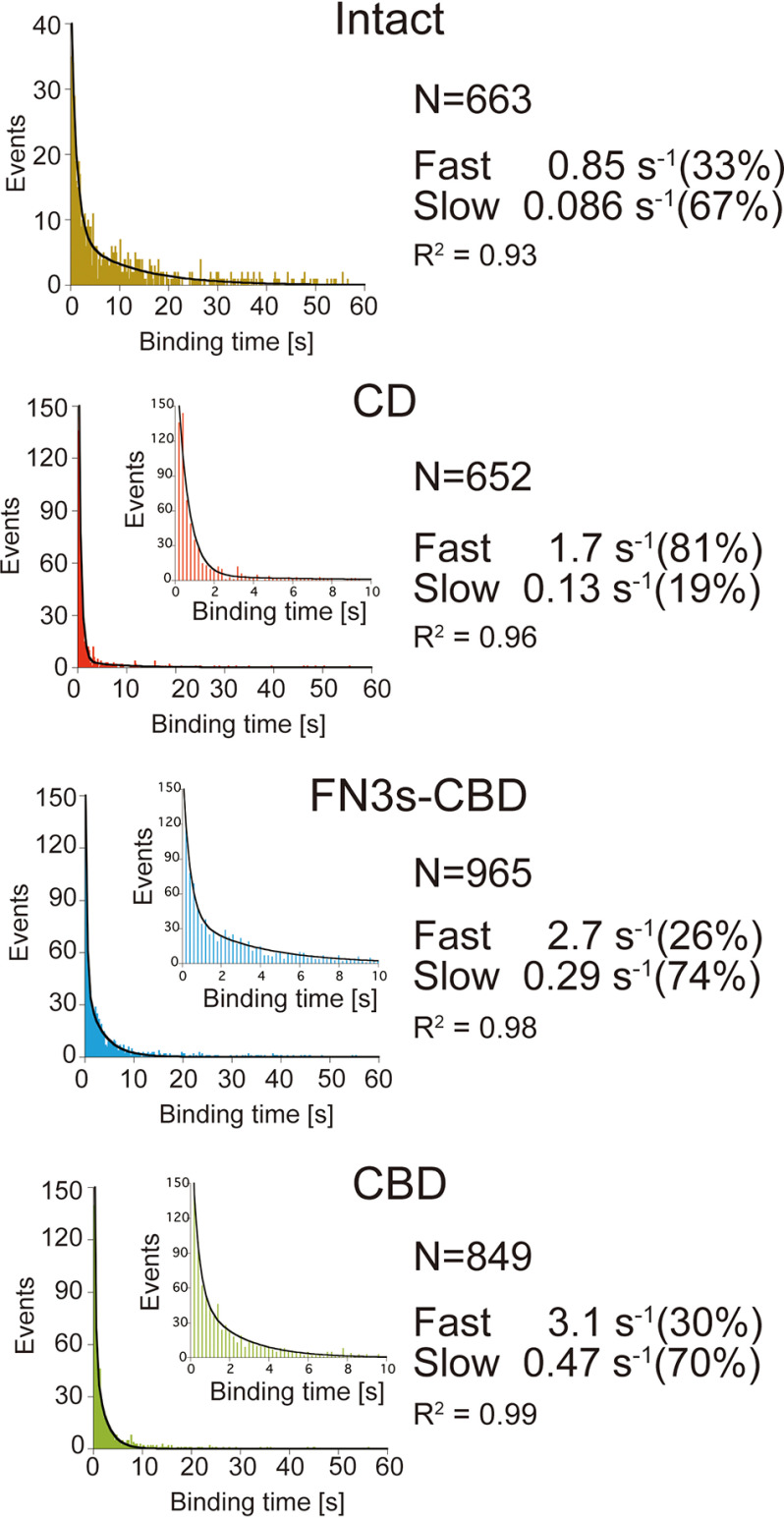
**Dissociation rate constants (*k*_off_) analysis of Intact, CD, FN3s-CBD, and CBD of CfCel6B.** Distributions of binding times were fitted by sum of two exponential decay functions. The values and ratios of the fast and slow components of the dissociation rate constant ($k_{\text{off}}^{\text{fast}}$ and $k_{\text{off}}^{\text{slow}}$) are shown on the *right*. The *N* values represent numbers of analyzed molecules.

Next, we analyzed distribution of binding time on cellulose surface. Distributions of binding times were better fitted by sum of two exponential decay functions than single exponential decay in Intact, FN3-CBD, and CBD (Fig. 4 and Fig. S1), as reported previously for TrCel6A (12). These results indicate that at least two different binding modes of the enzyme exist. For CD, although the *R*^2^ values were same for both fittings, the sum of the two exponential decay functions was used to estimate the fractions of two modes. Fast and slow components of dissociation rate constant ($k_{\text{off}}^{\text{fast}}$ and $k_{\text{off}}^{\text{slow}}$, respectively) for Intact were 0.85 and 0.086 s^−1^, respectively. The ratios of fast and slow components were 33 and 67% respectively. Those for CD were 1.7 s^−1^ (81%) and 0.13 s^−1^ (19%) and increased 1.5–2 times compared with those for Intact. In contrast, *k*_off_ values and ratios of fast components for FN3s-CBD and CBD were 2.7 s^−1^ (26%) and 3.1 s^−1^ (30%), respectively. In addition, those of slow components for FN3s-CBD and CBD were 0.29 s^−1^ (74%) and 0.47 s^−1^ (70%), respectively. These *k*_off_ values were comparable between FN3s-CBD and CBD but increased more than three times compared with those for Intact. These results indicated that cellulose-bound state of CfCel6B is stabilized by CD. We also found that CD showed much higher ratio of fast dissociation (81%) than that of slow dissociation (19%). This result was unique for CD among the four samples (*i.e.* Intact, CD, FN3s-CBD, and CBD), because the ratios of slow dissociation were almost 70% for the other three samples.

### Affinity and dissociation constant

The values of the binding rate constant corresponding to the fast and slow components ($k_{\text{on}}^{\text{fast}}$ and $k_{\text{on}}^{\text{slow}}$, respectively) were estimated from the *k*_on_ shown in Fig. 3 and the ratio of fast and slow components determined by the *k*_off_ analysis shown in Fig. 4 (Table 1). Then the values of the dissociation constant (*K_d_*) for the fast and slow components ($K_{d}^{\text{fast}}$ and $K_{d}^{\text{slow}}$, respectively) were calculated from the ratio of *k*_off_ to *k*_on_ (*k*_off_/*k*_on_). Among them, $K_{d}^{\text{slow}}$ for Intact showed the lowest value (3.0 × 10^−10^ m µm). This value was 20 times lower than that of $K_{d}^{\text{fast}}$ for Intact. The values of $K_{d}^{\text{fast}}$ or $K_{d}^{\text{slow}}$ for FN3s-CBD and CBD were comparable and less than 15 times higher than those of Intact. On the other hand, $K_{d}^{\text{slow}}$ for CD was 133 times higher than that of Intact because of the low value of $k_{\text{on}}^{\text{slow}}$. The difference of $K_{d}^{\text{fast}}$ values for CD and Intact was 20 times. These results indicate that CBD mainly contributes to the affinity of both fast and slow components of Intact.

**Table 1:** **Summary of binding-rate, dissociation-rate, and dissociation constants**

Sample	*k*_on_	Component	$k_{\text{off}}^{\text{fast}}$ or $k_{\text{off}}^{\text{slow}}$*^a^*		$k_{\text{on}}^{\text{fast}}$ or $k_{\text{on}}^{\text{slow}}$*^c^*	$K_{d}^{\text{fast}}$ or $K_{d}^{\text{slow}}$*^d^*
			Value	Ratio*^b^*		
	*m*^−*1*^ μ*m*^−*1*^*s*^−*1*^		*s*^−*1*^	%	*m*^−*1*^ μ*m*^−*1*^ *s*^−*1*^	*m* μ*m*
Intact	4.3 × 10^8^	Fast	0.85	33	1.4 × 10^8^	6.0 × 10^-9^
		Slow	0.086	67	2.9 × 10^8^	3.0 × 10^-10^
CD	1.7 × 10^7^	Fast	1.7	81	1.4 × 10^7^	1.2 × 10^-7^
		Slow	0.13	19	3.2 × 10^6^	4.0 × 10^-8^
FN3s-CBD	2.0 × 10^8^	Fast	2.7	26	5.2 × 10^7^	5.2 × 10^-8^
		Slow	0.29	74	1.5 × 10^8^	2.0 × 10^-9^
CBD	1.5 × 10^8^	Fast	3.1	30	4.5 × 10^7^	6.9 × 10^-8^
		Slow	0.47	70	1.1 × 10^8^	4.5 × 10^-9^

*^a^*The $k_{\text{off}}^{\text{fast}}$ and $k_{\text{off}}^{\text{slow}}$ are the fast and slow components of the dissociation rate constant, obtained by the fitting of the distribution of binding time distribution (Fig. 4) with a double exponential decay function.

*^b^*The ratios of fast and slow components were calculated from the ratio of the area of each fitted exponential decay function.

*^c^*The *k*_on_ values determined in Fig. 3 were further divided into $k_{\text{on}}^{\text{fast}}$ and $k_{\text{on}}^{\text{slow}}$ by using the ratio of fast and slow components of *k*_off_.

*^d^*The $K_{d}^{\text{fast}}$ and $K_{d}^{\text{slow}}$ values were calculated as $k_{\text{off}}^{\text{fast}}$/$k_{\text{on}}^{\text{fast}}$ and $k_{\text{off}}^{\text{slow}}$/$k_{\text{on}}^{\text{slow}}$, respectively.

### Translational rate and processivity

**Figure 5.**
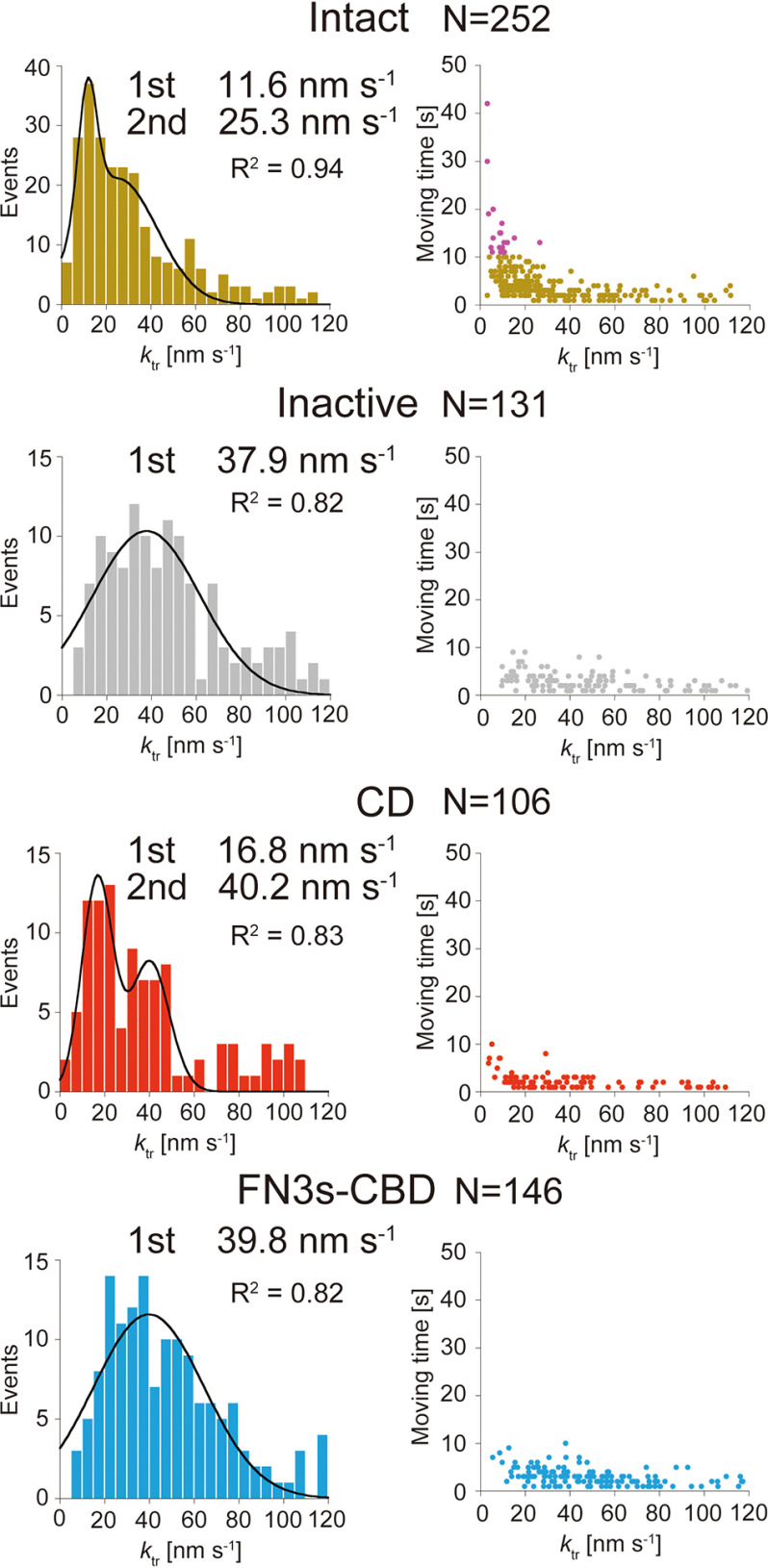
**Translational rate (*k*_tr_) and moving time analyses of Intact, Inactive, CD, and FN3s-CBD.** *Left panels*, distributions of *k*_tr_ fitted by Gaussian functions. Peak values of distributions are shown. *Right panels*, plots of *k*_tr_ *versus* moving time. For Intact, molecules moved more than 10 s are shown in *purple*. The *N* values represent numbers of analyzed molecules.

**Figure 6.**
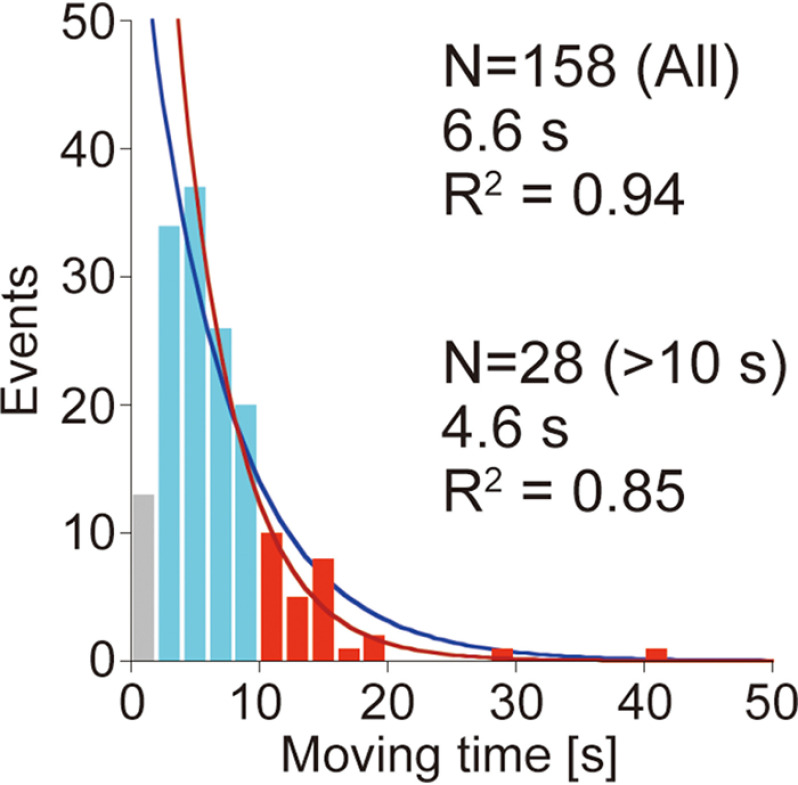
**Distribution of moving time.** Distribution of moving time for Intact was fitted by single exponential decay functions. *Blue* and *red lines* are fittings with the whole range and using only the range more than 10 s, respectively. The first bin was excluded from fittings. The *N* values represent numbers of analyzed molecules.

Next, translational rate (*k*_tr_) was measured from the distance between first and last positions of movement and moving time (Fig. 5). We used higher laser power density (0.28 µW µm^−2^) and lower frame rate (1 fps) than those for binding and dissociation analyses, to achieve higher localization precision (4.5 ± 1.5 and 4.6 ± 1.4 nm for the *x* and *y* axes, respectively). Distributions of *k*_tr_ for Intact and CD could be fitted with sum of two Gaussians (*R*^2^ values were 0.94 and 0.83, respectively). Peak values for Intact were 11.6 and 25.3 nm s^−1^, and those for CD were 16.8 and 40.2 nm s^−1^, respectively. On the other hand, distributions of *k*_tr_ for Inactive and FN3s-CBD could be fitted with a single Gaussian, and the peak values were 37.9 and 39.8 nm s^−1^, respectively (*R*^2^ values were both 0.82). Some Intact molecules moved more than 10 s, and all of these molecules showed lower *k*_tr_ near the first peak (Fig. 5, *right* and *top panel*). On the other hand, in other samples, no molecule moved more than 10 s (Fig. 5, *right panel*). These results strongly suggest that Intact molecules that showed a long moving time (>10 s) correspond to those moving processively, and Intact molecules that showed a short moving time (<10 s) are a mixture of those moving processively and diffusing on the cellulose surface. Therefore, the distribution of moving times for Intact was separately fitted to all ranges except for the first bin or the ranges longer than 10 s (Fig. 6). The time constant of moving time for the former was 6.6 s (*R*^2^ = 0.94) and that for the latter was 4.6 s (*R*^2^ = 0.85). These values were both shorter than the values for TrCel6A (7.7 s in both fittings) previously reported (12).

### Crystal structure of CfCel6B CD

**Figure 7.**
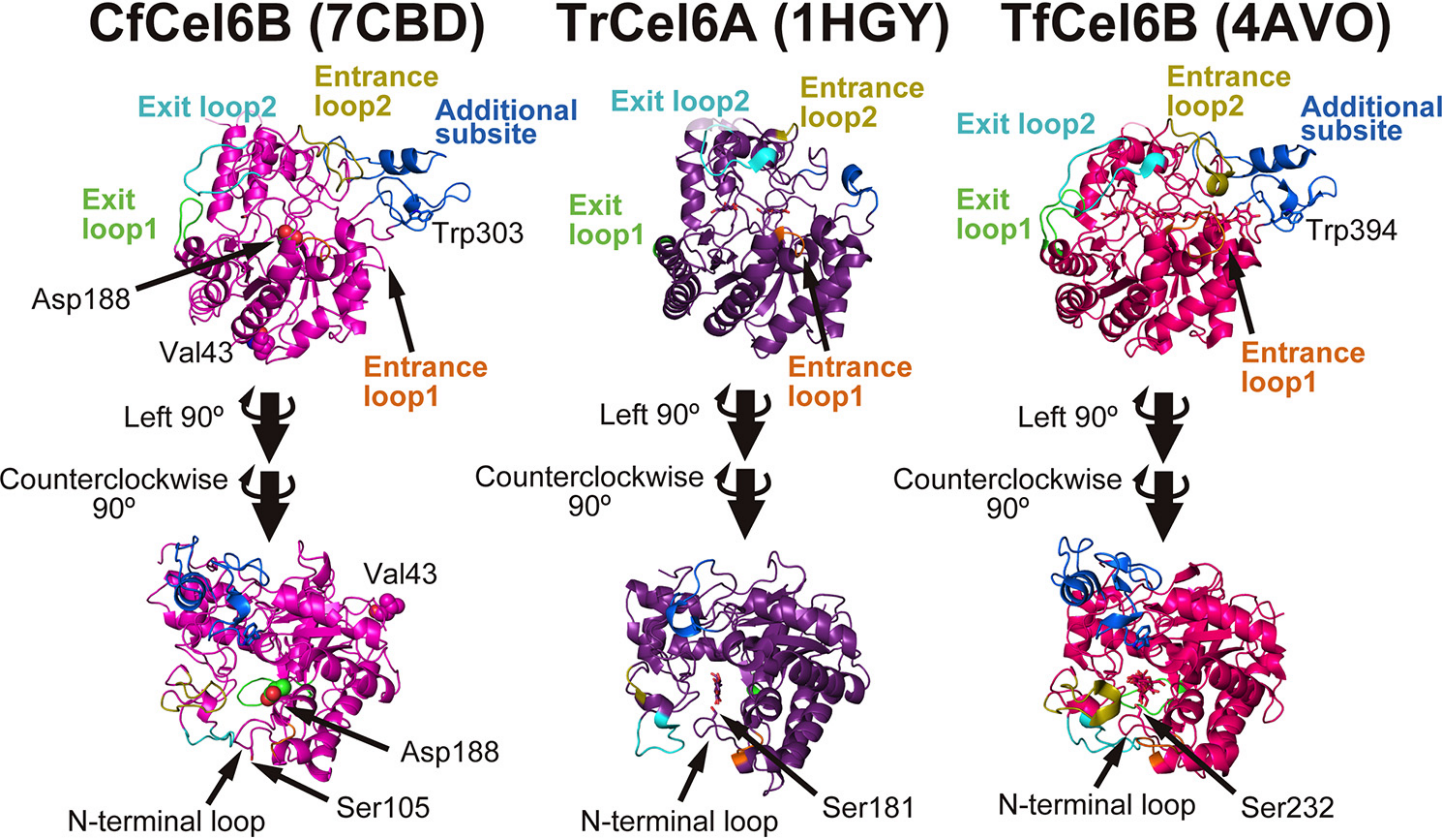
**Structural comparison of bacterial and fungal GH6 CDs.** *Left panel*, crystal structure of apo CfCel6B CD (PDB code 7CBD). Entrance loops 1 and 2 are shown in *orange* and *yellow*, and exit loops 1 and 2 are *green* and *cyan*, respectively. Loops constructing additional subsite are shown in *blue*. Val^43^ and Asp^188^ of CfCel6B, which are mutated to cysteine for fluorescent labeling and to alanine for inactivation, respectively, are shown as *spheres*. *Middle* and *right panels*, crystal structures of TrCel6A CD (PDB code 1HGY) and TrCel6B CD (PDB code 4AVO) shown from same directions and viewpoints. The active serine residues of three enzymes and the ligands in 1HGY and 4AVO are shown by *stick model*.

We solved a crystal structure of CfCel6B CD with 1.3 Å resolution (Table S1), to clarify the structural difference between CfCel6B and TrCel6A CDs (Fig. 7). The structure of CfCel6B CD was modeled by SWISS-MODEL server based on a GH6 CBH from a bacterium *Thermobifida fusca* (TfCel6B; PDB code 4AVO) and was used as a template of molecular displacement (13). In the determined crystal structure of CfCel6B CD, we confirmed that the Val^43^, which is close to the N terminus of CfCel6B (without signal peptide) and mutated to Cys in the single-molecule fluorescence imaging, was located in the opposite side of the catalytic site.

In the crystal structure, CfCel6B has additional substrate-binding site (subsite) constructed by Trp^303^ at plus side, the same as TfCel6B (Fig. 7, *right panel*). This additional subsite is stabilized by two loops (Fig. 7, *left panel*, shown in *blue*) which are not found in TrCel6A (Fig. 7, *middle panel*). Near the product-binding site, a pair of exit loops has been also found in CfCel6B and TfCel6B. The exit loop 1 (shown in *green*) was capping the end of the substrate-binding tunnel. This exit loop 1 of CfCel6B is 4 amino acids shorter than that of TfCel6B, indicating that the tunnel of CfCel6B is more open than that of TfCel6B. In addition, the exit loop 2 (shown in *cyan*) of CfCel6B showed more open conformations compared with that of TfCel6B.

The conformation of the N-terminal loop of CfCel6B was more open compared with those of TrCel6A and TfCel6B. The serine residue (Ser^105^, Ser^181^, and Ser^232^ in CfCel6B, TrCel6A, and TfCel6B, respectively), which is thought to be important to form a hydrogen bond network among the water molecules and catalytic residues, exists on the N-terminal loop (18). The Ser^105^ and the N-terminal loop of CfCel6B, crystallized without ligand, stayed outside of the cleft. In contrast, the serine residues interact with the ligand, and the N-terminal loops face to inside of the cleft in the structures of TrCel6A and TfCel6B. The conformational change of the N-terminal loop caused by the interaction with ligand has been reported previously for the GH6 CBH from a basidiomycete (19).

### Comparison of GH6 CDs from fungi and bacteria

**Figure 8.**
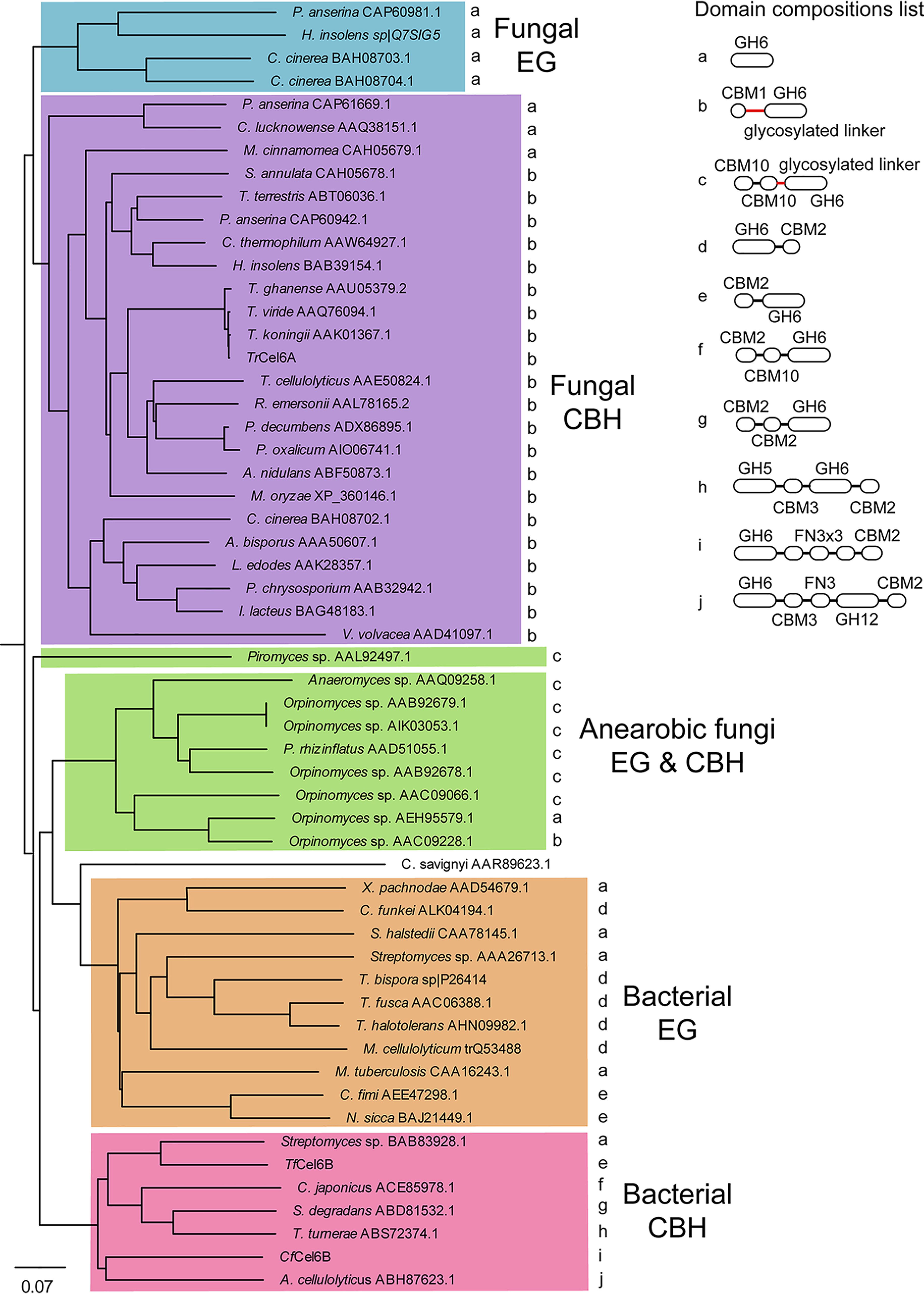
**Phylogenetic tree of GH6 CD from bacteria and fungi and their domain compositions.** GH6 enzymes in CAZy database were analyzed. GH6 regions were determined by homology modeling by SWISS-MODEL server and aligned depending on their structures using MODELLER. CBH and EG were classified according to the description in CAZy or original papers. Domain compositions were extracted from NCBI database or results of homology modeling. Glycosylated linkers are shown in *red*.

To discuss the relationship between the structure and function of CD, we compared all CDs of GH6 enzymes listed in CAZy database (RRID:SCR_012909), except the enzymes with only patent information or including unknown residues in the sequence. By the structural alignment of X-ray crystal structures and homology modeled structures of CD, five groups were identified (Fig. 8). In the phylogenetic tree diagram, bacterial and fungal cellulases were clearly separated. Interestingly, EGs and CBHs from aerobic bacteria and fungi formed different groups, but those of anaerobic fungi were mixed.

Each group showed characteristic domain compositions, although the phylogenetic tree was prepared based on the sequences of only CD (Fig. 8, *right panel*). For example, fungal EGs did not have CBD, and many of CBHs were constructed by CD and CBM1-CBD with serine- or threonine/proline-rich, glycosylated linker region. Many anaerobic fungal cellulases had a glycosylated linker and two CBM10-CBDs that show ∼6 times weaker affinity than that of CBM2-CBD (20). On the other hand, bacterial EGs had three types of domain compositions. One had only CD, and the other two had CD with CBM2-CBD in the N or C terminus. Furthermore, bacterial CBHs showed completely different compositions. CBM2-CBD was the major component, although CBM3-CBD and CBM10-CBD were also found. In addition, two of them had an additional CD domain classified into GH5 or GH12 EGs.

Given the domain composition lists of CBHs from fungi and bacteria, the CBM1 with the glycosylated linker is the common domains for fungal CBHs. The CBM2 is the common domain for bacterial CBH, but the FN3s is not. Another interesting point is the order of domains. The fungal CBHs had CBM1 on the N terminus of CD. Although bacterial CBHs did not show clear order, further characterizations of GH6 CBHs from other bacteria is required to draw a conclusion.

## Discussion

In this study, we found that Intact CfCel6B exhibits similar *k*_cat_ to TrCel6A (2.4 and 2.8 s^−1^ for CfCel6B and TrCel6A, respectively) (Fig. 2), although CfCel6B has largely different domain composition from that of TrCel6A (12). On the other hand, the *K_m_* value for Intact CfCel6B (0.51 mg ml^−1^) was much lower than that for TrCel6A (2.7 mg ml^−1^), indicating that the affinity of CfCel6B to the crystalline cellulose is higher than that of TrCel6A. To understand the differences in the mechanisms of crystalline cellulose hydrolysis by these enzymes, here we quantitatively compare the kinetic parameters of elementary reaction steps such as binding (*k*_on_), translational movement (*k*_tr_ and processivity), and dissociation (*k*_off_) determined in the present and previous studies (12).

Our single-molecule fluorescence imaging enables direct estimations of the *k*_on_ and *k*_off_ of processive cellulases separately to understand which parameter mainly affects the affinity to the crystalline cellulose (Figs. 3 and 4). For both CfCel6B and TrCel6A, we found fast and slow components that correspond to the bindings on hydrophilic and hydrophobic crystal surfaces of the cellulose, respectively (Table 1) (12). Considering that cellulose hydrolysis will occur on hydrophobic, high-affinity crystal surface, the slow component is more relevant to the productive binding, although only a few binding events will lead to the hydrolysis. The values of $k_{\text{on}}^{\text{slow}}$ for Intact CfCel6B and TrCel6A were comparable and 2.9 × 10^8^ m^−1^ μm^−1^ s^−1^ (Fig. 3 and Table 1) and 2.3 × 10^8^ m^−1^ μm^−1^ s^−1^ (12), respectively. The values of $k_{\text{off}}^{\text{slow}}$ for Intact CfCel6B and TrCel6A were 0.086 and 0.10 s^−1^, respectively (Fig. 4 and Table 1) (12). Thus, the values of $k_{\text{off}}^{\text{fast}}$ or $k_{\text{off}}^{\text{slow}}$ between Intact CfCel6B and TrCel6A are similar. However, the ratio of slow dissociation component for CfCel6B was 67% and much higher than that for TrCel6A (30%). The higher ratio of the slow dissociation component indicates that CfCel6B more specifically binds to the hydrophobic surface than TrCel6A. This difference is one of the reasons that CfCel6B showed a lower *K_m_* value than TrCel6A. However, quantitative comparison is not easy, because direct measurement of the *k*_on_ for productive binding was difficult because of the low frame rate and localization precisions, similar to the case for our recent single-molecule fluorescence imaging of chitinase A from *Serratia marcescens* (21).

To understand role of the CD in the binding and dissociation of CfCel6B and TrCel6A on the cellulose surface, we also measured *k*_on_ and *k*_off_ for CfCel6B CD (Figs. 3 and 4) and solved its crystal structure (Fig. 7). Overall structure of CfCel6B CD was similar to that of the TrCel6A CD except for the additional subsite and loops. Although Trp^303^ in additional subsite is exposed to the solvent, the values of *k*_on_ and *k*_off_ for CfCel6B CD were similar to those for TrCel6A CD measured in our previous study (12). In addition, the ratio of $k_{\text{off}}^{\text{slow}}$ for CfCel6B CD (19%, Table 1) was not largely different from that for TrCel6A (28%). Therefore, the binding and dissociation are not largely affected by the additional subsite and loops of CfCel6B CD.

The most crucial domain for binding and dissociation of CfCel6B is CBD (Figs. 3 and 4). In the present study, the CBD showed 10 times higher *k*_on_ than CD, and the value was more than one-third of that for Intact (Table 1). These results clearly indicate that CBD has a critical role in the initial interaction of CfCel6B with crystalline cellulose. On the other hand, comparison of $k_{\text{off}}^{\text{slow}}$ or $k_{\text{off}}^{\text{fast}}$ between CD and CBD showed that binding of CD to hydrophobic or hydrophilic surface of crystalline cellulose is more stable than those of CBD (Fig. 4 and Table 1). However, the ratio of slow dissociation component for CD was 19% and much lower than that for CBD (70%), which was close to the value for Intact (67%). Therefore, stability of binding for Intact is a result of the cooperation between CD and CBD, but the specificity of binding to the hydrophobic surface arises from the binding by CBD. The CBD of CfCel6B is a member of type A CBM2, which is specific to cellulose and has a flat surface with aromatic residues (22). Binding of CBD in this group is expected to be driven by an increase of entropy (23), and this would be a reason for the high specificity of CfCel6B CBD to the hydrophobic surface of crystalline cellulose (Table 1). In case of TrCel6A CBD, the ratio of slow dissociation component was only 30% in our previous report (12). Because TrCel6A CBD belongs to CBM1, this result indicates that CBM2 is more specific to the hydrophobic surface than CBM1. Higher affinity of CBM2 was reported by Tomme *et al.* (24) using the CBM1 of the GH7 CBH from *T. reesei* and CBM2 of a GH10 xylanase from *C. fimi*. The preference of CBD binding to the hydrophobic surface has been reported previously by Nimlos *et al.* (25). In their simulation, within few hundred nanoseconds, CBM1 moved to the hydrophobic surface from the hydrophilic surface. Similar events might also occur for CfCel6B CBD, although we could not resolve such a short time event in our single-molecule fluorescence imaging. As a conclusion, both CBDs of CfCel6B and TrCel6A have a role to lead the CD to the hydrophobic surface of crystalline cellulose, on which an accessible chain end exists.

Another large difference between TrCel6A and CfCel6B is the linker region. The contribution of the glycosylated linker of fungal cellulase to the binding on the cellulose surface has been reported previously (12, 14). On the other hand, in the present study, FN3s of CfCel6B do not contribute to the interaction with the cellulose surface, because FN3s-CBD showed similar *k*_on_ and *k*_off_ values with CBD (Table 1). Although FN3s are one of a common domains in bacterial glycoside hydrolases, such as polygalacturonosidase, chitinase, pullulanase, amylase, and cellulase (26), FN3s were not conserved in bacterial GH6 CBHs (Fig. 8). Recently, Valk *et al.* (27) proposed that the FN3s in bacterial GHs work as stable linkers connecting functional domains (CD and CBD) to keep the relative orientation and distance. Although our results support this idea, FN3-like domain of chitinase A from *S. marcescens* has a function as a binding domain on chitin (28). Therefore, we need to carefully compare the sequence, structure, and function of FN3s in other cases.

Other important parameters we need to compare are *k*_tr_ and processivity. The *k*_tr_ for Intact CfCel6B showed two slow and fast components with peak values of 11.6 and 25.3 nm s^−1^, respectively (Fig. 5). We attribute the slow component of Intact to processive movement coupled with the hydrolysis of cellulose chain, because the value of *k*_tr_ is similar to the moving velocity (12.7 nm s^−1^) observed by high-speed atomic force microscopy (29), and Inactive showed only fast component (37.9 nm s^−1^). From this result, the hydrolytic activity of productively bound Intact CfCel6B molecule can be estimated to be 11.6 s^−1^, because the length of product cellobiose is ∼1 nm. The large gap between the activities estimated from biochemical analysis (2.4 s^−1^; Fig. 2), and single-molecule analysis (11.6 s^−1^) is presumably due to the low fraction of productive binding, as previously demonstrated for chitinase A from *S. marcescens* (30). From the values described above, only 20% of the Intact molecules is estimated to be productively bound even at high substrate concentration. This low ratio is caused by the limited numbers of the accessible chain ends on the surface of crystalline cellulose. Slow and fast movements in the translational movement on crystalline cellulose have been also observed in TrCel6A, and *k*_tr_ for slow and fast components were 8.8 and 34.9 nm s^−1^, respectively (12). The fast movements correspond to surface diffusion without hydrolysis of cellulose chain as reported previously on the CBM2 using fluorescence recovery after the photobleaching method (31).

Furthermore, an interesting difference was found between CDs of CfCel6B and TrCel6A. The slow component for CD, which was not found in TrCel6A, has been observed in CfCel6B (Fig. 5). This result strongly suggests that CfCel6B CD has higher processivity than TrCel6A CD by the additional subsite and loops observed in the crystal structure (Fig. 7). Another possibility is that additional Trp increases efficiency to catch a cellulose chain end to from a productive complex. On the other hand, the value of processivity for CfCel6B Intact, estimated from *k*_tr_ (11.6 nm s^−1^; Fig. 5), moving time (4.6 s; Fig. 6), and size of the product (cellobiose, 1.0 nm), was 53, which is smaller than 68, the value for TrCel6A Intact estimated from the same analysis (12). These results strongly suggest that contribution of glycosylated linker and CBD of TrCel6A to the processivity is larger than that of FN3s-CBD of CfCel6B. Our results also indicate that not only the structure of CD but also the linker and CBD are important for the processivity.

Domain compositions of GH6 cellulases clearly showed different tendencies between fungi and bacteria. When the phylogenetic tree of GH6 CD was prepared, groups of fungal EG and CBH, bacterial EG and CBH, and anaerobic fungal cellulase were separated as expected (Fig. 8). However, this phylogenetic tree also indicates an interesting relationship between the domain compositions and the function of CD. For instance, fungal CBH basically has glycosylated linker and CBM1-CBD to achieve high-affinity binding and high processivity for efficient degradation of crystalline cellulose. In contrast, bacterial CBHs have large variety in combinations of domains and length of linker regions. The common properties among them are CBM2-CBD with high affinity and CD with a long substrate-binding tunnel (and high processivity, presumably). These two domains, important for crystalline cellulose degradation, were highly conserved except for the CBH from *Streptomyces* sp. Therefore, bacterial CBHs seem to compensate for weak interaction of nonglycosylated linker region with crystalline cellulose surface by strong binding of CBM2-CBD and high processivity of CD. As a result, bacterial and fungal GH6 CBHs have a similar function: processive hydrolysis of the crystalline cellulose from the nonreducing end. The phylogenetic tree analysis suggests that the bacterial and fungal CBHs have been generated by a divergent evolution from an ancestor, but converged functionally. The glycosylated linker and CBM1-CBD of fungal GH6 CBHs are not common among the bacterial GH6 CBHs, and the CBM2-CBD of bacterial CBHs is rarely observed in eukaryote either, although the catalytic domain has the same fold. Therefore, the two groups may have evolved independently, and both obtain the binding function using the unique ways for fungi or bacteria to degrade the crystalline cellulose.

Recently, a cellulase containing a GH6 CBH from *Reconcilibacillus cellulovorans* (RcCelC) has been reported (32). Domain composition of this enzyme is GH6-GH5-CBM3, and GH6 CD is similar to the bacterial one. Although RcCelC does not have CBM2, it contains Ser-Pro-Thr-rich linkers between GH6 and GH5, and GH5 and CBM3, respectively. In their study, glycosylation of RcCelC has been confirmed by Periodic acid–Schiff staining. Recently, glycosylation of bacterial proteins also has been considered to be common, especially in pathogenic bacteria (33). The cellulases with glycosylated linker and bacterial GH6 CBH are one of the next interesting targets to analyze the elementary steps of the reaction and processivity by using the single-molecule imaging analysis.

## Experimental procedures

### Mutant design and gene preparation

The CfCel6B gene without signal peptide (Ala^41^ to Gly^872^ of CfCel6B WT) was amplified with primers including NcoI (CCATGG) and HindIII (AAGCTT) recognition sites for forward and reverse primers, respectively, and ligated to pET27b vector. As a result, one methionine residue was added in the N terminus of enzyme. The gene of CfCel6B CD (Met plus Ala^41^–Thr^483^ of CfCel6B WT) for crystallization was amplified with primers containing NcoI sand HindIII recognition sites. Six histidine residues and the stop codon were connected to the codon corresponding to Thr^483^ and ligated with pET27b after digestion by NcoI and HindIII. The genes of CfCel6B V43C was amplified by same forward primer additionally containing V43C mutation (GTC to TGC). The reverse primer was prepared at RsrII recognition site (1514 bp downstream from Ala^41^). Part of CfCel6B gene was swapped with amplified fragment. For CD-V43C, the same forward primer and reverse primers including the codons for FaXa protease recognition site (IEGRFGG: ATCGAAGGCCGCTTTGGCGGC) between the codons of Thr^483^ and the His_6_ tag were used. CfCel6B V43C/D188A gene was prepared by additional a pair of primers, which has an 18-bp overlap region, for the D188A mutation. The two fragments of DNA from Ala^41^ to Ala^188^ and Ala^188^ to the RsrII site (near Val^546^) were mixed and amplified with the same primers for CfCel6B V43C. The flagrant was ligated with the plasmid of CfCel6B V43C treated by NcoI and RsrII. The gene of FN3-CBD (Val^484^–Gly^872^) V484C was amplified by primers with NcoI and HindIII sites. The start codon, His_6_ tag, and FaXa recognition site were added to the N terminus of the protein. The gene was swapped with CfCel6B. CBD (Thr^766^–Gly^872^) T766C was also amplified with similar sets of primers. The gene was also ligated to pET27B. Primester GXL polymerase (Takara) was used for all of PCRs. Restriction enzymes were purchased from NEB. PCR products were purified by agarose gel electrophoresis and Wizard® SV Gel and PCR clean-up system (Promega) according to the manufacturer's instruction. Ligations of DNA fragments were achieved by Mighty Mix kit (Takara). All of ligated plasmids were transformed into Tuner (DE3) (Merck Millipore) by electroporation using MicroPulser (Bio-Rad) according to the setting for *E. coli* transformation. 50 µl of transformed competent cell was mixed with 200 µl of SOC medium and incubated at 37 °C for 1 h. All of suspension were spread on agarose LB plate containing 25 µg ml^−1^ kanamycin and incubated for a night at 37 °C. Three or four colonies were cultivated in 10 ml of LB medium containing 25 µg ml^−1^ kanamycin at 37 °C and 300 rpm for 16 h. The plasmids were purifed from the cells by FastGene plasmid mini kit (NIPPON Genetics). Whole sequences of genes were verified, and the plasmids were stored in −30 °C.

### Expression, purification, and Cy3 labeling of protein samples

Plasmids were transformed into *E. coli* Tuner (DE3) by electroporation in the same way above. Single colonies of one-fourth of the plate were inoculated in 10 ml of LB medium with 25 µg ml^−1^ kanamycin and incubated at 37 °C and 200 rpm for 1 h. 3 ml of preculture medium was added in 50 ml of Overnight Express instant LB medium (Novagen) containing 25 µg ml^−1^ of kanamycin and incubated at 25 °C and 130 rpm for a night. The cell was harvested by centrifuge at 3000 × *g* for 10 min at 4 °C. Harvested cell was stored in −80 °C until purification.

For the purification of CfCel6B WT, Intact, and inactive, ∼7 g of cell was suspended in 70 ml of 100 mm Tris-HCl, pH 8.0, containing 100 mm sodium chloride. The cells were disrupted by sonication for 15 min on ice. Suspension was mixed with 30 µl of Benzonase (Merck Millipore) and 710 µl of 2 m magnesium chloride, and precipitant was removed by centrifuge at 8,000 × *g* for 10 min and 30,000 × *g* for 10 min sequentially at 37 °C. Two times the amount of 3 m ammonium sulfate was added to supernatant and centrifuged at 8,000 × *g* 10 min. Supernatant was loaded on cellulose column equilibrated with 1 m ammonium sulfate (34). Unbound protein was washed out by 1 m ammonium sulfate, and bound protein was eluted by milliQ water. Purity of proteins were analyzed by SDS-PAGE, and the fractions containing the ∼80-kDa protein were collected and concentrated by 30-kDa cut Vivaspin 20 column at 6000 × *g*. Buffer was changed to 20 mm Tris-HCl, pH 7.5, by Econo-Pack 10DC column (Bio-Rad). The enzyme was loaded on the Toyoperl DEAE 650-S (Tosho) and eluted by the linear gradient of sodium chloride from 0 to 300 mm. Target proteins were concentrated by Vivaspin. CfCel6B WT was further loaded into YMC-Pack Diol-200G (YMC) and eluted by 20 mm sodium phosphate, pH 7.0, with 100 mm sodium chloride. Other free cysteine mutants were reduced by 10 mm DTT for 2 h at 25 °C before being put into a size-exclusion column. Reduced protein was loaded to YMC-Pack Diol-200G and eluted by 20 mm sodium phosphate, pH 7.0, with 100 mm sodium chloride. The protein in the fraction showed peak absorption at 280 nm chromatogram was reacted with five times higher moles of Cy3-maleimide for a night at room temperature. Unreacted Cy3 was removed by Econo-Pack 10DC column with 20 mm sodium phosphate, pH 7.0, with 100 mm sodium chloride. Labeled enzyme was concentrated, and labeling ratio was calculated. Molecular extinction coefficient of 131,650 m^−1^ cm^−1^ at 280 nm was used for CfCel6B, and those of 12,000 m^−1^ cm^−1^ at 280 nm and 150,000 m^−1^ cm^−1^ at 550 nm were used for Cy3. Purified protein was kept at −80 °C after flash freezing with liquid nitrogen.

*E. coli* cells expressing CfCel6B CD, FN3-CBD, and CBD were disrupted and centrifuged by the same method without Benzonase and magnesium chloride. Supernatant was loaded on nickel–nitrilotriacetic acid–agarose column (Qiagen) and washed by 50 mm sodium phosphate, pH 7.0, with 100 mm sodium chloride. The column was washed by the buffer containing 20 mm imidazole, and target proteins were eluted by 50 and 100 mm imidazole-containing buffer. Collected proteins were concentrated to 200 μl by ultracentrifuge, and 4 µl of 100 mm calcium chloride and 20 µl of 1 mg ml^−1^ FaXa protease (NEB) were added to sample. After incubation at 23 °C for a night, and target protein was reduced by 10 mm DTT at 25 °C for 1 h. Reduced protein was injected to HPLC equipped with YMC-Pack Diol-200G. Target protein was eluted by 50 mm sodium phosphate, pH 7.0, with 100 mm sodium chloride. For labeling, the procedures were same to those of CfCel6B Intact and inactive. Molecular coefficient of CD was ε280 = 71,930 m^−1^ cm^−1^, those of FN3-CBD and CBD were ε280 = 59,720 m^−1^ cm^−1^ and ε280 = 29,850 m^−1^ cm^−1^. CfCel6B CD for crystallization was purified without FaXa treatment and reduction. Molecular coefficient of CD for crystallization was ε280 = 1.49 mg^−1^ ml cm^−1^. TrCel6A Intact was the same sample used in the previous report (12).

### Activity measurement of enzymes

Purified 0.5 μm of CfCel6B WT, Intact, and inactive were reacted with 1 mg ml^−1^ of crystalline cellulose I_α_ in 100 µl of 100 mm sodium acetate buffer, pH 5.0, at 30 °C. After 1 h of incubation, 100 µl of sodium hydroxide was added to stop the reaction. Suspension was centrifuged at 15,000 × *g* for 10 min, and 120 µl of supernatant was mixed with same volume of 4-hydroxybenzoic acid hydrazide (PAHBAH) solution (35). The mixture was heated at 95 °C for 10 min, and absorbance at 405 nm of 220 µl of solution was taken by 96-well plate reader. Standard curve was prepared using glucose, and activity was calculated as a normalized value by time and enzyme concentration.

To determine the turnover and affinity of CfCel6B WT and Intact to crystalline cellulose I_α_, 0.1 μm of CfCel6B WT and Intact were incubated with 0.3, 1.0, 3.0, and 5.0 mg ml^−1^ of crystalline cellulose in 100 mm sodium acetate buffer, pH 5.0, at 25 °C for 2 min. Supernatant as collected after centrifuge and concentration of products were analyzed by HPLC (11).

### Observation of binding, dissociation, and translational movement

Cover glass (24 mm × 32 mm, Matsunami Glass) was incubated in 10 m potassium hydroxide for a night and washed by milliQ water. For the single-molecule observation, we used annular illumination single-molecule fluorescence microscopy with EM-CCD camera (Andor) (12). 20 μl of 0.2 mg ml^−1^ crystalline cellulose I suspension was coated on the glass at 3000 rpm by spin coater (Mikasa) as described previously (12). For analyses of binding rate and dissociation rate constants, 20 µl of 50 pm Intact, FN3-CBD, 100 pm CBD, or 600 pm CD was dropped on the glass. The power density of a 532-nm laser was set at 0.14 µW µm^−2^, and the frame rate was 5 fps. The rate constant of photobleaching for Cy3 conjugated with CfCel6B was 0.053 ± 0.002 s^−1^ (or time constant of 18.9 ± 0.7 s) under this observation condition. Crystalline cellulose microfibrils were stained by 10 µl of 10 nm TrCel6A-S386C-Cy3, and the movie was overlaid with the image to analyze the molecules bound on the cellulose. Lengths of the crystalline cellulose microfibrils were measured by using ImageJ as previously described (36). Binding rates were corrected by the labeling ratio. For the analysis of translational movement and *k*_tr_, the laser power density of 0.28 µW µm^−2^ and frame rate of 1 fps were used to improve the localization precision. Under this observation condition, localization precisions in the *x* and *y* axes were 4.5 ± 1.5 and 4.6 ± 1.4 nm, respectively. Time constant of photobleaching for Cy3 conjugated with CfCel6B was 14.9 ± 0.6 s.

### X-ray crystal structure analysis of CfCel6B CD

1 μl of 10 mg ml^−1^ purified CfCel6B CD was mixed with 1 µl of 21% PEG3350 and 10 mm sodium chloride in 100 mm sodium acetate buffer, pH 5.0, on the sitting-drop plate (griner). The drop was equilibrated with 100 µl of 21% PEG3350 and 10 mm sodium chloride in 100 mm sodium acetate buffer, pH 5.0, for a week at 20 °C. Formed rod-like crystal was soaked in 40% PEG3350, 10 mm sodium chloride, and 100 mm sodium acetate, pH 5.0. Diffraction of 1.0 Å X-ray was measured from 0 to 360° with 0.5° oscillation. Diffraction spots were observed up to 1.3 Å resolution, the diffraction images were processed by HKL2000, and the phase was determined by Phaser in Phenix suite (37). The template for molecular replacement was prepared by the SWISS-MODEL server using TfCel6B (PDB code 4AVO) as a template for modeling (38). Structural refinement and model editing was done by Phenix refine and Coot (39). CDs of CfCel6B, TfCel6B, and TrCel6A were compared and visualized by PyMOL.

### Comparison of GH6 enzymes from bacteria and fungi

Amino acid sequences of GH6 cellulase classified as characterized enzymes in CAZy database were downloaded. Some enzymes containing X residues or reported only as patent were rejected. Homology model structures of GH6 CD were prepared by SWISS-MODEL server with default settings (38). GH6 CDs were aligned by multiple structural alignment function in MODELLER (40). Phylogenetic tree was calculated by Clustal Omega server using the neighbor-joining method (41). The tree file was visualized by Figtree. Domain configurations were determined according to NCBI, Uniprot database, and results of modeling.

## Data availability

The structure presented in this article has been deposited into the Protein Data Bank (PDB) with the following ID: 7CBD. All remaining data are contained within the article.

## Supplementary Material

Supporting Information
