# Supplementary material for: Domain architecture divergence leads to functional divergence in binding and catalytic domains of bacterial and fungal cellobiohydrolases
Source: J Biol Chem. 2020 Aug 18;295(43):14606–17. doi: 10.1074/jbc.RA120.014792 (PMC7586223; doi:10.1074/jbc.RA120.014792)
Supplement: Supporting Information [file supp_295_43_14606__index.html]

Domain architecture divergence leads to functional divergence in binding and catalytic domains of bacterial and fungal cellobiohydrolases — Single-molecule fluorescence imaging of bacterial cellulase — Supporting Information 

# Domain architecture divergence leads to functional divergence in binding and catalytic domains of bacterial and fungal cellobiohydrolases

## Supporting Information

- Supporting Information (to be published online) - Table S1 and Figure S1
