# Supplementary material for: Domain architecture divergence leads to functional divergence in binding and catalytic domains of bacterial and fungal cellobiohydrolases
Source: J Biol Chem. 2020 Aug 18;295(43):14606–17. doi: 10.1074/jbc.RA120.014792 (PMC7586223; doi:10.1074/jbc.RA120.014792)
Supplement: Supporting Information [file supp_RA120.014792_161473_2_supp_582830_qf7yhy.pdf]

Supplementary Information for:

Domain architecture divergence leads to functional divergence in binding and catalytic domains of bacterial and fungal cellobiohydrolases

Akihiko Nakamura[1]*, Daiki Ishiwata[2,3], Akasit Visootsat[2,3], Taku Uchiyama[4], Kenji Mizutani[5], Satoshi Kaneko[6], Takeshi Murata[5], Kiyohiko igarashi[4] and Ryota Iino[2,3]*

[1]Department of Applied Life Sciences, Faculty of Agriculture, Shizuoka University, 836 Ohya, Suruga-ku, Shizuoka, Shizuoka 422-8529, Japan

[2]Department of Functional Molecular Science, School of Physical Sciences, SOKENDAI (The Graduate University for Advanced Studies), Hayama, Kanagawa 240-0193, Japan

[3]Institute for Molecular Science, National Institutes of Natural Sciences, 5-1 Higashiyama Myodaijicho, Okazaki, Aichi 444-8787, Japan

[4]Department of Biomaterials Sciences, Graduate School of Agricultural and Life Sciences, University of Tokyo, Tokyo 113-8657, Japan

[5]Department of Chemistry, Graduate School of Science, Chiba University, 1-33 Yayoi-cho, Inage, Chiba 263-8522, Japan

[6]Department of Subtropical Biochemistry and Biotechnology, Faculty of Agriculture, University of the Ryukyus, Nishihara, Okinawa 903-0213, Japan

*Correspondence: Akihiko Nakamura (aki-naka@shizuoka.ac.jp) and Ryota Iino (iino@ims.ac.jp)

Table S1. Summary of X-ray diffraction data and refinement of model

CfCel6B CD (PDB ID: 7CBD)	
Data collection	
Beam line	KEK BL5A
Wavelength (Å)	1.0
Resolution range (Å)	50.0 - 1.30 (1.32 - 1.30)
Space group	C121
Unit Cell (Å)	107.7, 49.4, 71.0
Unit Cell (°)	90, 96.36, 90
Total reflections	368253 (17842)
Unique reflections	81834 (3965)
Multiplicity	4.5 (4.5)
Completeness (%)	89.5 (87.2)
Mean I / sigma (I)	39.2 (10.9)
Wilson B-factor ($Å^2$)	8.14
R-merge	0.038 (0.149)
Refinement	
R-work	0.1256 (0.1235)
R-free	0.1559 (0.1780)
RMS (bond) (Å)	0.006
RMS (angles) (°)	0.863
Ramachandran favored (%)	97.5
Ramachandran allowed (%)	2.5
Ramachandran outliers (%)	0
Clash score	2.32
Average B-factor	13.8

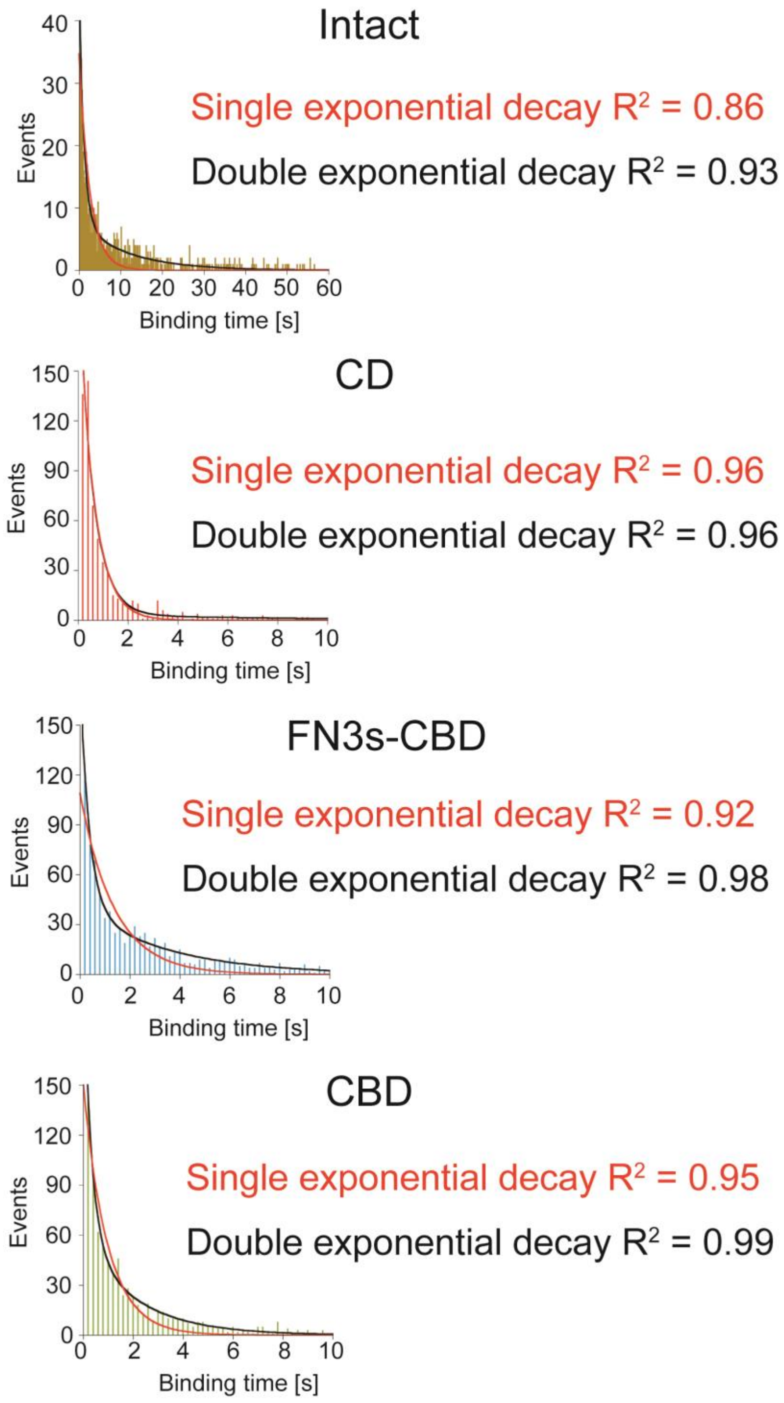

Supplementary Figure S1. Comparison of single exponential decay and double exponential decay functions for fitting to the distribution of binding time. The data shown in Figure 4 were fitted with single exponential and double exponential decay functions and goodness of the fitting were compared. Fitting curves of single exponential decay are shown in red, and those of double exponential decay are shown in black.
